# Fulminant Hepatic Failure Attributed to Ackee Fruit Ingestion in a Patient with Sickle Cell Trait

**DOI:** 10.1155/2012/739238

**Published:** 2012-10-08

**Authors:** Dianne E. Grunes, Irini Scordi-Bello, Matthew Suh, Sander Florman, Jonathan Yao, Maria Isabel Fiel, Swan N. Thung

**Affiliations:** ^1^Department of Pathology, Mount Sinai School of Medicine, One Gustave L. Levy Place, P.O. Box 1194, New York, NY 10029, USA; ^2^Office of the Chief Medical Examiner, 520 First Avenue, New York, NY 10016, USA; ^3^Department of Surgery, Mount Sinai School of Medicine, One Gustave L. Levy Place, P.O. Box 1194, New York, NY 10029, USA

## Abstract

We report a case of fulminant liver failure resulting in emergent liver transplantation following 3 weeks of nausea, vomiting, and malaise from Jamaican Vomiting Sickness. Jamaican Vomiting Sickness is caused by ingestion of the unripe arils of the Ackee fruit, its seeds and husks. It is characterized by acute gastrointestinal illness and hypoglycemia. In severe cases, central nervous system depression can occur. In previous studies, histologic sections taken from patients with Jamaican Vomiting Sickness have shown hepatotoxicity similar to that seen in Reye syndrome and/or acetaminophen toxicity. We highlight macroscopic and microscopic changes in the liver secondary to hepatoxicity of Ackee fruit versus those caused by a previously unknown sickle cell trait. We discuss the clinical variables and the synergistic hepatotoxic effect of Ackee fruit and ischemic injury from sickled red blood cells, causing massive hepatic necrosis in this patient.

## 1. Background

Ackee fruit has been associated with toxicity since the late 19th century [1]. It has since been known that the toxins are hypoglycin A and hypoglycin B [2]. Hypoglycin A and B are found in the immature arils of the fruit and in the seeds and husks of the plants throughout maturation. The mechanism of toxicity is not entirely understood, but they are known to interfere with the metabolism of fatty acids, leading to hypoglycemia and acidosis [3]. However, vomiting and central nervous system depression can be seen in the absence of hypoglycemia and with only mild acidosis [4]. In addition to glycogen depletion in the liver, a histologic appearance of toxic liver injury similar to that seen in acetaminophen overdose has been documented [5]. The response to the toxins is dose dependent and gastrointestinal symptoms begin within 6–48 hours, with complete recovery typical within the week [6]. In fatal cases, death typically occurs within 48 hours of ingestion [4, 6].

Ackee is not sold in the United States. However, it has been consumed for centuries in some parts of the world. It is the national dish of Jamaica and is a large part of the diet of Haitian, West African, and Jamaican foods [7]. Most fatalities reported have been of children and malnourished adults, who are particularly susceptible to the hypoglycemic effects of the toxin [4, 6, 8, 9]. Most adult cases are of vomiting sickness followed by recovery [9]. The only treatment for this illness is largely supportive [10]. 

## 2. Case Presentation

This was a case of a 61-year-old African American woman in previously good health with no known medical conditions. She presented to the Emergency Department at St. Barnabas on June 20, 2011, after being found unconscious by her family after 3 weeks of nausea, malaise, and frequent bouts of vomiting thought by her primary care physician to be due to hepatitis A. On admission, she was in fulminant hepatic failure with hepatic encephalopathy. Initial workup was negative for a definitive etiology; her IgG anti-hepatitis A was positive, but IgM was negative. The remainder of her viral hepatitis serology was negative as was her toxicology screen for drugs of abuse and acetaminophen. Her social history was negative for alcohol or drug use. Family history revealed a brother with sickle cell trait. She denied taking supplements or herbal medications. Travel history was positive for a recent trip to Haiti and Jamaica, 3 weeks prior to presentation. On her return, she went to a family cookout where she consumed Ackee fruit as part of the main course. Her symptoms began shortly thereafter. 

She was transferred to The Mount Sinai Medical Center on June 23rd for transplant evaluation. She was considered particularly high risk because she was a Jehovah's Witness and declined blood transfusion. On admission, she was mildly anemic with a hematocrit (Hct) of 33.9% (reference range 34.0–47.0%) and in fulminant hepatic failure; International Normalized Ratio (INR) was 4.8, albumin 2.2 g/dL (reference range 3.5–4.9), aspartate aminotransferase (AST) 620 U/L (reference range 1–50), alanine aminotransferase (ALT) 478 U/L (reference range 1–53), and total bilirubin 24.0 mg/dL (reference range 0.1–1.2). According to patient's prior wishes and family wishes, an advance directive was signed refusing packed red blood cells, fresh frozen plasma, and platelet transfusion. She had accepted cryoprecipitate, recombinant factor 7, erythropoietin, hemodilution, and CellSaver usage. While controversial, the transplant team agreed to list her for emergent transplantation. She received factor 7 prior to the procedure and tolerated the transplantation well without technical complication and with minimal blood loss without the need for vasopressor support. 

After the procedure her Hct was 20%, INR 4.3, AST 655 U/L, and ALT 1805 U/L. The posttransplant ultrasound showed patent vessels. She required resuscitation as well as vasopressor support, albumin boluses, and cryoprecipitate. The next day, her serum lactate level increased to 23 mmol/L (reference range 0.5–1.6) with worsening acidosis (pH 6.87). Her liver tests continued to deteriorate dramatically: AST 15,309 U/L, ALT 5,767 U/L, total bilirubin 6.6 mg/dL, and INR 12.2 despite additional doses of factor 7, cryoprecipitate, and albumin. After one successful code, her code status was changed to “Do Not Resuscitate.” She expired 6 hours later. An autopsy was requested by the family; the case was accepted by the Medical Examiner. 

## 3. Pathology of the Native Liver

### 3.1. Gross Examination

**Figure 1:**
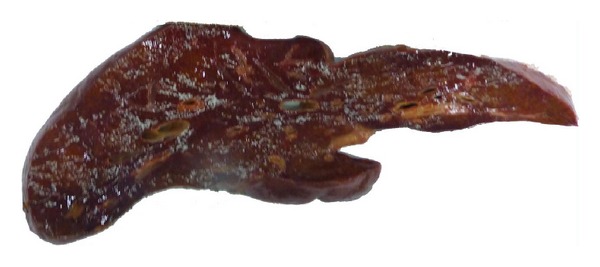
Gross photograph of the native liver. The specimen weighs 645 g and measures 23 × 15 × 4 cm. Cross section through greatest dimension of the liver shows a shrunken and hemorrhagic liver indicative of parenchymal loss.

The liver weighs 645 grams and appears shrunken with wrinkled capsule attributed to parenchymal collapse. Cut surfaces show prominent portal areas with collapsed dark tan parenchyma. The gross findings are consistent with massive hepatic necrosis (Figure 1).

### 3.2. Microscopic Examination

**Figure 2:**
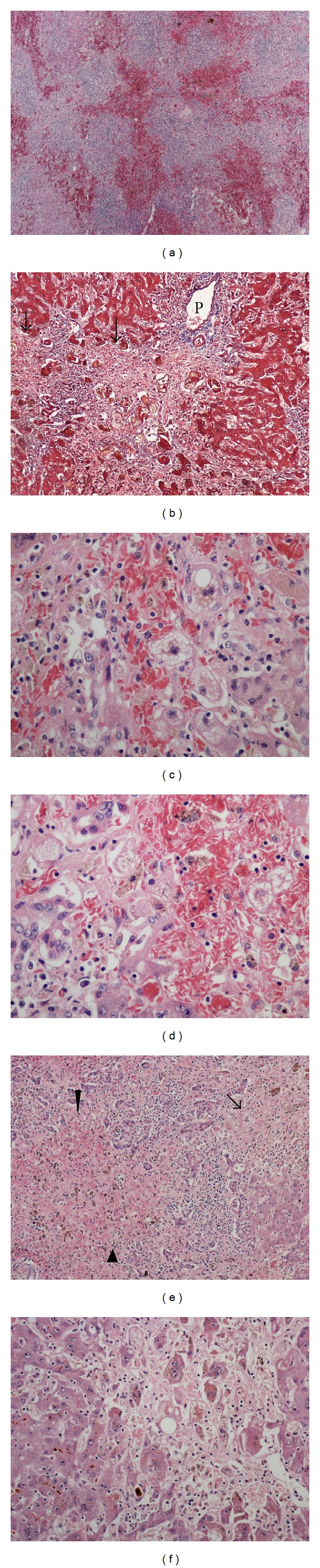
Histologic sections from the explanted liver. (a) The portal tracts are close together separated by hemorrhagic areas with hepatocyte loss (H&E, ×20). (b) Trichrome stain highlights a portal tract (P), but pale areas of parenchymal collapse (arrows) are unstained (×100). (c) Hepatocytes with microvesicular steatosis and sickled red blood cells (H&E, ×200). (d) Ductular hepatocytes and inflammatory cells adjacent to an area with hepatocyte loss and sickled cells (H&E, ×200). (e) Periportal ductular hepatocytes in an area of confluent necrosis. Microvesicular steatosis (arrow) and sickled cells of varying ages (arrow heads) are also highlighted in this section (H&E, ×40). (f). Regenerating hepatocytes adjacent to an older area of parenchymal loss. Note multinucleated giant hepatocytes with cholestasis in a largely hypocellular background with some macrophages (H&E, ×200).

There are bridging and multiacinar necroses with approximation of adjacent portal tracts and severe centrilobular congestion and hemorrhage (Figure 2(a)). No fibrous septa are identified on trichrome stain (Figure 2(b)). In areas with central to central bridging necrosis, periportal hepatocytes are preserved while in areas with multiacinar collapse, ductular hepatocytes occupy the periportal regions (Figure 2(c)). Regenerative changes, that is, 2 cell thick cords and multinucleation are seen in the periportal hepatocytes. Mild inflammation is present in necrotic areas. The inflammatory cells consist of lymphocytes and macrophages. Cholestasis and small fat droplets (microvesicular steatosis) are seen in the neighboring hepatocytes (Figure 2(d)). There are sickled red blood cells of various ages lodged in the sinusoids (Figures 2(e) and 2(f)). The findings strongly suggest that there has been ongoing sickling of red blood cells, which results in ischemic damage in addition to the toxic injury. 

### 3.3. Autopsy Findings

Histological sections taken from various organs at postmortem examination show changes of hypovolemic shock. The liver has centrilobular coagulative necrosis without evidence of vascular thrombosis or rejection. Kidneys have acute tubular necrosis with few bile casts. A small number of sickled red blood cells are found in the lungs. 

## 4. Discussion

Drug- or toxin-induced liver injury (DILI) can range from mild to severe liver damage that may result in massive hepatic necrosis requiring liver transplantation. The diagnosis is only made after having excluded viral and autoimmune diseases. The case that is being reported here is that of hepatic injury likely resulting from ingestion of a toxin found in Ackee fruit. Ackee toxin, unfortunately, is metabolized quickly [4] and could not be quantified on postmortem examination or from stored blood from her hospital admission. The toxic effect was compounded by ischemic injury from sickling of red blood cells in a patient with previously unknown sickle cell trait. She had never been tested, although her deceased brother was a known sickle cell trait, as per information given by family members to the office of Medical Examiners. The absence or presence of other cases with gastrointestinal distress was not known to the family. This could be due to differential amounts of ingestion, tolerance, or due to a reporting bias. Pathological examination revealed acute liver injury with bridging and multiacinar necroses. Microvesicular steatosis seen in some hepatocytes has been described in Ackee fruit toxicity from hypoglycin A metabolic injury. Hypoglycin A also causes ischemia in the vulnerable centrilobular area. The forceful vomiting characteristic of Jamaican Vomiting Sickness (JVS) causes dehydration, which can precipitate a sickling crisis in those with sickle cell disease [3, 11–13].

The different ages of the sickled cells lodged in the sinusoids suggest an ongoing sickling of red blood cells in-vivo with a more recent peri- and postoperative ischemia resulting in areas of fresh hemorrhage. The extent and on-going nature of the hepatic injury explains the prolonged time course; the gastrointestinal symptoms worsened over three weeks instead of resolving after one week. While the extent of hepatic damage and the prolongation of the time course can be explained by toxic injury combined with sickle cell trait, there are differences in her presentation from that typically seen in JVS that cannot be attributed to her sickle cell trait. On presentation she was encephalopathic but not hypoglycemic, although hypoglycemia is a classic sign of JVS [7–10]. However, in the distribution of symptoms in a study of 60 patients with JVS, only 19% had documented hypoglycemia, while 97% had vomiting and 43% had loss of consciousness [4]. Furthermore, the only previously described case of Ackee fruit poisoning in the United States did not present with hypoglycemia [9]. Though exceedingly rare in the United States, JVS is endemic to Jamaica and Haiti [4, 8]. This case is unique not only because of the severity of the illness but because it has not been previously described in a patient with sickle cell trait. This is surprising as sickle cell trait reaches up to 30% of the population in areas of West Africa, Haiti, and Jamaica, where Ackee fruit is a dietary staple and where most cases of JVS have been documented [4]. Though this may have been because of paucity of autopsies in these areas, it remains a puzzling point. Hepatic failure secondary to vaso-occlusive crisis in sickle cell disease has also been documented and is often fatal [11, 14–16]. 

This patient most likely suffered from sickle cell trait and not sickle cell disease. This distinction is made based upon the patient's lack of a known blood cell dyscrasia up to the age of 61. 

In conclusion, given the patient's history and histopathologic findings, the most likely suspect for the toxic injury is ingestion of Ackee fruit. The toxic hepatocyte injury coupled with sickling-induced ischemic injury, ultimately acting synergistically to cause fulminant hepatic failure, previously not described.
